# Supplementary material for: Modeling glioblastoma heterogeneity as a dynamic network of cell states
Source: Mol Syst Biol. 2021 Sep 16;17(9):e10105. doi: 10.15252/msb.202010105 (PMC8444284; doi:10.15252/msb.202010105)
Supplement: Supplementary file 5 — Source Data for Figure 3 [file MSB-17-e10105-s001.zip › Figure3A_sourcedata/GSEA_3065/hallmarks_state3.GseaPreranked.1623416274744/HALLMARK_COMPLEMENT.html]

Details for gene set HALLMARK\_COMPLEMENT[GSEA]

|  || Dataset | state3 |
| Phenotype | NoPhenotypeAvailable |
| Upregulated in class | na\_pos |
| GeneSet | HALLMARK\_COMPLEMENT |
| Enrichment Score (ES) | 0.33223552 |
| Normalized Enrichment Score (NES) | 1.279978 |
| Nominal p-value | 0.05172414 |
| FDR q-value | 0.1386039 |
| FWER p-Value | 0.765 |
Table: GSEA Results Summary

  

Fig 1: Enrichment plot: HALLMARK\_COMPLEMENT      
 Profile of the Running ES Score & Positions of GeneSet Members on the Rank Ordered List

  

| PROBE | GENE SYMBOL | GENE\_TITLE | RANK IN GENE LIST | RANK METRIC SCORE | RUNNING ES | CORE ENRICHMENT || 1 | PLAT |  |  | 18 | 0.513 | 0.0390 | Yes |
| 2 | TIMP1 |  |  | 35 | 0.470 | 0.0747 | Yes |
| 3 | HSPA5 |  |  | 43 | 0.447 | 0.1096 | Yes |
| 4 | TFPI2 |  |  | 65 | 0.388 | 0.1382 | Yes |
| 5 | LRP1 |  |  | 152 | 0.309 | 0.1530 | Yes |
| 6 | LAMP2 |  |  | 154 | 0.309 | 0.1775 | Yes |
| 7 | PRCP |  |  | 166 | 0.303 | 0.2005 | Yes |
| 8 | CTSC |  |  | 226 | 0.272 | 0.2154 | Yes |
| 9 | ADAM9 |  |  | 229 | 0.272 | 0.2369 | Yes |
| 10 | TIMP2 |  |  | 230 | 0.271 | 0.2586 | Yes |
| 11 | COL4A2 |  |  | 232 | 0.271 | 0.2801 | Yes |
| 12 | CD46 |  |  | 357 | 0.229 | 0.2841 | Yes |
| 13 | CTSB |  |  | 392 | 0.219 | 0.2977 | Yes |
| 14 | DOCK10 |  |  | 395 | 0.219 | 0.3150 | Yes |
| 15 | CTSD |  |  | 398 | 0.219 | 0.3322 | Yes |
| 16 | CD59 |  |  | 713 | 0.168 | 0.3094 | No |
| 17 | CTSL |  |  | 735 | 0.166 | 0.3203 | No |
| 18 | FN1 |  |  | 973 | 0.140 | 0.3041 | No |
| 19 | CLU |  |  | 988 | 0.139 | 0.3136 | No |
| 20 | DGKH |  |  | 1182 | 0.123 | 0.3012 | No |
| 21 | CPM |  |  | 1215 | 0.120 | 0.3071 | No |
| 22 | CPQ |  |  | 1371 | 0.110 | 0.2980 | No |
| 23 | GNAI3 |  |  | 1417 | 0.107 | 0.3014 | No |
| 24 | LGMN |  |  | 1637 | 0.095 | 0.2837 | No |
| 25 | USP15 |  |  | 1689 | 0.093 | 0.2852 | No |
| 26 | RCE1 |  |  | 1791 | 0.087 | 0.2805 | No |
| 27 | KIF2A |  |  | 1899 | 0.081 | 0.2746 | No |
| 28 | DYRK2 |  |  | 1913 | 0.080 | 0.2795 | No |
| 29 | PIK3CA |  |  | 1919 | 0.080 | 0.2853 | No |
| 30 | RBSN |  |  | 2202 | 0.068 | 0.2582 | No |
| 31 | PCLO |  |  | 2292 | 0.065 | 0.2531 | No |
| 32 | C1R |  |  | 2407 | 0.060 | 0.2448 | No |
| 33 | GPD2 |  |  | 2606 | 0.053 | 0.2262 | No |
| 34 | ZEB1 |  |  | 2764 | 0.048 | 0.2119 | No |
| 35 | PSEN1 |  |  | 2814 | 0.046 | 0.2099 | No |
| 36 | LIPA |  |  | 2893 | 0.044 | 0.2045 | No |
| 37 | CTSO |  |  | 2920 | 0.043 | 0.2049 | No |
| 38 | VCPIP1 |  |  | 3079 | 0.039 | 0.1898 | No |
| 39 | SERPINE1 |  |  | 3090 | 0.039 | 0.1917 | No |
| 40 | USP14 |  |  | 3096 | 0.038 | 0.1942 | No |
| 41 | AKAP10 |  |  | 3212 | 0.035 | 0.1838 | No |
| 42 | IRF1 |  |  | 3457 | 0.029 | 0.1579 | No |
| 43 | IRF7 |  |  | 3530 | 0.027 | 0.1518 | No |
| 44 | USP8 |  |  | 3605 | 0.025 | 0.1453 | No |
| 45 | L3MBTL4 |  |  | 3692 | 0.023 | 0.1372 | No |
| 46 | GCA |  |  | 3924 | 0.017 | 0.1119 | No |
| 47 | GNB4 |  |  | 3971 | 0.016 | 0.1078 | No |
| 48 | CTSV |  |  | 4007 | 0.015 | 0.1050 | No |
| 49 | PLAUR |  |  | 4075 | 0.013 | 0.0983 | No |
| 50 | PLSCR1 |  |  | 4373 | 0.005 | 0.0644 | No |
| 51 | LAP3 |  |  | 4547 | -0.000 | 0.0444 | No |
| 52 | EHD1 |  |  | 4716 | -0.004 | 0.0253 | No |
| 53 | CASP4 |  |  | 4831 | -0.007 | 0.0127 | No |
| 54 | MMP15 |  |  | 4903 | -0.008 | 0.0052 | No |
| 55 | SH2B3 |  |  | 5212 | -0.016 | -0.0291 | No |
| 56 | CA2 |  |  | 5274 | -0.018 | -0.0348 | No |
| 57 | RAF1 |  |  | 5375 | -0.020 | -0.0447 | No |
| 58 | GMFB |  |  | 5412 | -0.021 | -0.0471 | No |
| 59 | PRDM4 |  |  | 5463 | -0.023 | -0.0511 | No |
| 60 | LTA4H |  |  | 5492 | -0.024 | -0.0524 | No |
| 61 | USP16 |  |  | 5641 | -0.028 | -0.0672 | No |
| 62 | SIRT6 |  |  | 5737 | -0.031 | -0.0757 | No |
| 63 | RNF4 |  |  | 5743 | -0.031 | -0.0738 | No |
| 64 | XPNPEP1 |  |  | 5786 | -0.032 | -0.0761 | No |
| 65 | PREP |  |  | 6373 | -0.052 | -0.1396 | No |
| 66 | STX4 |  |  | 6484 | -0.056 | -0.1479 | No |
| 67 | DUSP6 |  |  | 6502 | -0.056 | -0.1453 | No |
| 68 | ANXA5 |  |  | 6522 | -0.057 | -0.1429 | No |
| 69 | CASP7 |  |  | 6580 | -0.060 | -0.1447 | No |
| 70 | RABIF |  |  | 6600 | -0.061 | -0.1421 | No |
| 71 | IRF2 |  |  | 6602 | -0.061 | -0.1374 | No |
| 72 | CEBPB |  |  | 6863 | -0.074 | -0.1615 | No |
| 73 | CSRP1 |  |  | 6916 | -0.077 | -0.1614 | No |
| 74 | FYN |  |  | 6924 | -0.077 | -0.1560 | No |
| 75 | CASP3 |  |  | 6969 | -0.079 | -0.1548 | No |
| 76 | GNG2 |  |  | 7035 | -0.083 | -0.1556 | No |
| 77 | MSRB1 |  |  | 7259 | -0.097 | -0.1736 | No |
| 78 | CALM1 |  |  | 7459 | -0.113 | -0.1876 | No |
| 79 | GRB2 |  |  | 7509 | -0.118 | -0.1838 | No |
| 80 | RHOG |  |  | 7526 | -0.119 | -0.1761 | No |
| 81 | FDX1 |  |  | 7571 | -0.125 | -0.1712 | No |
| 82 | PDP1 |  |  | 7720 | -0.142 | -0.1769 | No |
| 83 | PPP2CB |  |  | 7824 | -0.155 | -0.1764 | No |
| 84 | PSMB9 |  |  | 7835 | -0.156 | -0.1651 | No |
| 85 | PPP4C |  |  | 7996 | -0.184 | -0.1688 | No |
| 86 | MT3 |  |  | 8329 | -0.265 | -0.1860 | No |
| 87 | CALM3 |  |  | 8355 | -0.276 | -0.1669 | No |
| 88 | GNAI2 |  |  | 8368 | -0.280 | -0.1459 | No |
| 89 | LGALS3 |  |  | 8522 | -0.347 | -0.1358 | No |
| 90 | GNB2 |  |  | 8612 | -0.415 | -0.1128 | No |
| 91 | S100A13 |  |  | 8652 | -0.457 | -0.0808 | No |
| 92 | ATOX1 |  |  | 8705 | -0.526 | -0.0447 | No |
| 93 | PFN1 |  |  | 8732 | -0.624 | 0.0022 | No |
Table: GSEA details [plain text format]

  

Fig 2: HALLMARK\_COMPLEMENT: Random ES distribution      
 Gene set null distribution of ES for **HALLMARK\_COMPLEMENT**
